# Randomized controlled trial of a 12-week digital care program in improving low back pain

**DOI:** 10.1038/s41746-018-0076-7

**Published:** 2019-01-07

**Authors:** Raad Shebib, Jeannie F Bailey, Peter Smittenaar, Daniel A Perez, Gabriel Mecklenburg, Simon Hunter

**Affiliations:** 1 Hinge Health, Inc, San Francisco, CA USA; 2 Department of Rehabilitation Services, Kaiser Permanente, San Francisco, CA USA; 3 Department of Orthopaedic Surgery, University of California, San Francisco, CA USA

**Keywords:** Health care, Medical research

## Abstract

Low back pain (LBP) is the leading cause of disability throughout the world and is economically burdensome. The recommended first line treatment for non-specific LBP is non-invasive care. A digital care program (DCP) delivering evidence-based non-invasive treatment for LBP can aid self-management by engaging patients and scales personalized therapy for patient-specific needs. We assessed the efficacy of a 12-week DCP for LBP in a two-armed, pre-registered, randomized, controlled trial (RCT). Participants were included based on self-reported duration of LBP, but those with surgery or injury to the lower back in the previous three months were excluded. The treatment group (DCP) received the 12-week DCP, consisting of sensor-guided exercise therapy, education, cognitive behavioral therapy, team and individual behavioral coaching, activity tracking, and symptom tracking – all administered remotely via an app. The control group received three digital education articles only. All participants maintained access to treatment-as-usual. At 12 weeks, an intention-to-treat analysis showed each primary outcome—Oswestry Disability Index (*p* < 0.001), Korff Pain (*p* < 0.001) and Korff Disability (*p* < 0.001)—as well as each secondary outcome improved more for participants in the DCP group compared to control group. For participants who completed the DCP (per protocol), average improvement in pain outcomes ranged 52-64% (Korff: 48.8–23.4, VAS: 43.6–16.5, VAS impact on daily life: 37.3–13.4; *p* < 0.01 for all) and average improvement in disability outcomes ranged 31–55% (Korff: 33.1–15, ODI: 19.7–13.5; *p* < 0.01 for both). Surgical interest significantly reduced in the DCP group. Participants that completed the DCP had an average engagement, each week, of 90%. Future studies will further explore the effectiveness of the DCP for long-term outcomes beyond 12 weeks and for a LBP patient population with possibly greater baseline pain and disability. In conclusion, the DCP resulted in improved LBP outcomes compared to treatment-as-usual and has potential to scale personalized evidence-based non-invasive treatment for LBP patients.

## Introduction

According to the World Health Organization, low back pain (LBP) is the leading cause of disability worldwide with a global prevalence of 7.2%,^1^ affecting 4 in 5 individuals in their lifetime.^2,3^ Clinical diagnosis of LBP is difficult due to its multifactorial etiology and in turn, 90% of cases are designated as non-specific with no clear underlying cause.^4,5^ Given the uncertainties in diagnoses, localized LBP is treated with a broad variety of interventions including activity modification, physical therapy, pain medication, and spine injections. If symptoms do not improve, surgical intervention may be recommended. In the US, the economic costs of LBP are the highest in the world exceeding $100B per year^6^ and this is in part due to the high rates of surgical intervention.^7^ Health systems are not equipped to manage this growing population affected by LBP.

Patients pursuing non-invasive treatments have better outcomes for reducing disability and returning to work compared to those pursuing surgical intervention.^8^ In an evidence-based guideline, the American College of Physicians recommends to first pursue non-pharmacological conservative treatments for LBP because they are deemed less harmful.^9^ While exercise, rehabilitation, and cognitive behavioral therapy are among the most effective non-pharmacological conservative care treatments for ameliorating LBP symptoms, implementations of such care from a traditional clinical model has, so far, revealed inconsistent results.^9,10^ This is likely due to the high degree of patient engagement, commitment, and self-management needed to adhere and complete these time-intensive at-home treatment plans. The amount of patient engagement in a treatment plan is shown to directly relate to health outcomes,^11^ and is often an overlooked component in otherwise promising interventions.

Digital health technology can provide care for a large population and improve outcomes for non-invasive treatments by allowing providers to monitor adherence and activate patients to engage in their recovery. A digital therapy approach can integrate multiple conservative care channels while also tracking outcomes and providing biofeedback. The utilization of self-regulatory tools such as biofeedback as an engagement tool in non-specific LBP rehabilitation has been shown to promote greater than 80% adherence.^12^ Biofeedback enables patients to better learn how to voluntarily control and track therapeutic exercise by converting physical movement into meaningful visual and auditory cues.^13^ Biofeedback is believed to help patients gain awareness of their movement physiology and learn to self‐regulate and even challenge themselves to make progress in response to the real-time feedback.^13^

Surprisingly, results from prior randomized controlled trials of digital intervention on managing LBP with conservative care are largely unconvincing^14^ with only one prior study demonstrating a positive effect from a 3-week cognitive behavioral therapy digital intervention.^15^ Beyond the variability in theoretical underpinnings behind prior digital intervention studies, other issues may include the passive dissemination of content to patients and not assessing patient engagement. A digital care program (DCP) similar to the program tested here has recently been shown to be effective in alleviating knee pain outcomes and intent for surgery.^16,17^ The conservative care components of this unique DCP, including aerobic exercise, sensor-guided physical therapy-like exercises, patient education, and cognitive behavioral therapy, are known to be effective in treating LBP.^18–22^

In addition, the inclusion of personal health coaching, education, and group support are aimed at enhancing patient engagement in self-management of their symptoms.

In this study we assessed the efficacy of a 12-week DCP for LBP in a two-armed, pre-registered, randomized, controlled trial (RCT). Participants randomized into the treatment group received the 12-week DCP, consisting of sensor-guided exercise therapy, education articles, cognitive behavioral therapy, team discussions, activity tracking, symptom tracking, and 1-on-1 coaching, all from their home through a dedicated app on a complementary tablet computer. Participants randomized into the control group received three digital education articles only, and all participants maintained access to treatment-as-usual. that can include physician visits, pain medication, diagnostic imaging, and potential recommendations for later injections and/or surgery. Based on evidence of the potential for non-invasive therapies for treating LBP, we hypothesized that strong engagement with these multi-model conservative care approaches would improve pain and disability scores (primary outcomes), and subject understanding of their condition and their interest in surgery (secondary outcomes), compared to the control group. These outcomes as well as the eligibility criteria were registered prior to the initiation of the study (ISRCTN #42338218).

## Results

### Study population

Table 1 describes the demographics and screening data for the 177 participants randomized in the RCT. The average participant was 43 years old (SD: 11), slightly overweight (mean (SD) body mass index: 26 (4) kg/m^2^), and reasonably active (Godin activity score of 39). We observed no statistically significant difference in the gender ratio between groups (two-sided test of proportions, chi-squared = 1.70, *p* = 0.19). Nearly all participants were convinced that the DCP could help them either avoid surgery altogether (97%) or at least delay surgery (99%). The rate of opioid use in this population was 9%. A minority (12%) received some type of surgery on the back prior (>3 months) to starting the DCP, though participants still actively rehabilitating from surgery were excluded from the study. About 1 in 3 experienced pain not only in the lower back but also upper back (27%) and/or neck (32%). We observed a difference in prevalence of upper back pain (two-sided test, chi-squared = 4.2, *p* = 0.04). However, including upper back pain as covariate in the regression analyses did not change the results, hence this difference is not discussed further.

**Table 1:** Demographics of the control and treatment groups

	Treatment group (DCP)	Control group	All participants
Number of participants	113	64	177
Age in years, mean (SD)	43 (11)	43 (12)	43 (11)
Body-mass index (kg/m^2^), mean (SD)	26 (5)	26 (4)	26 (4)
Female, %	37%	48%	41%
physical therapy-like exercise at screening^a^, %	39%	50%	43%
Godin activity score^b^, mean (SD)	38 (32)	40 (27)	39 (30)
Hours sedentary per day, mean (SD)	5.9 (3.3)	5.4 (2.9)	5.8 (3.2)
Think DCP can help delay surgery, %	99%	100%	99%
Think DCP can help avoid surgery, %	95%	100%	97%
Taking opioids, %	10%	8%	9%
Self-efficacy^c^, mean (SD)	10.0 (3.7)	10.2 (3.3)	10.1 (3.6)
Healthcare visits for LBP in 12 weeks prior to screening, *n* (SD)	1.8 (3.5)	1.5 (3.0)	1.7 (3.3)
Back surgery > 3 months ago, %	12%	12%	12%
Experience neck pain, %	32%	33%	32%
Experience upper back pain, %	33%	17%	27%
STarT^d^ low risk, %	46%	45%	46%
STarT medium risk, %	35%	39%	37%
STarT high risk, %	19%	16%	18%

*SD* standard deviation

^a^Positive answer to the question “Do you currently do any physical therapy-style exercises?”

^b^Composite score, 24 indicates “active”, 14-23 indicates “Moderately active”, and <14 indicates “Insufficiently active/sedentary”^35^

^c^Health self-efficacy assessment, scores from 0 (no self-efficacy) to 15 (high self-efficacy)^36^

^d^STarT Back Screening Tool, risk of persistent pain^37^

Lastly, we observed no statistically significant differences in baseline scores for any of the primary and secondary outcomes (two-sided tests; all *p* ≥ 0.10).

### Participant flow

**Fig. 1:**
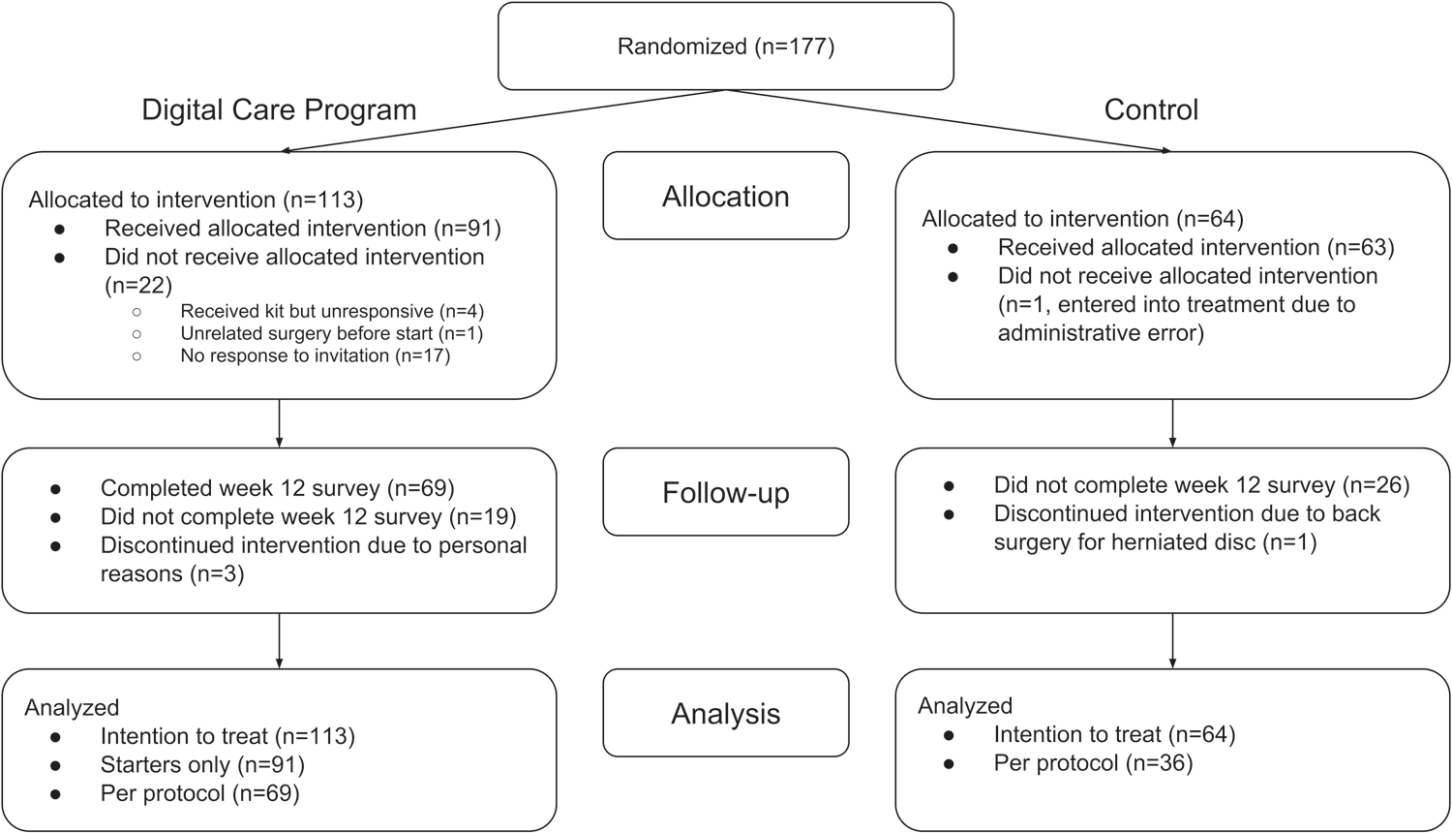
CONSORT flow diagram

Figure 1 represents a CONSORT flow diagram. As noted in the Methods, we used an uneven allocation ratio such that 113 (64%) participants entered DCP treatment, and 64 (36%) entered control. A number of participants were lost prior to the start of the DCP, when participants might have changed their mind about participating without communicating intent to withdraw during the remote screening and onboarding process. Nonetheless, these participants are included in the intention-to-treat analysis.

### Engagement

A major benefit of digital programs is the ability to track each participant’s daily engagements with the DCP over the 12 week program. Engagement with the program in the DCP group is summarized in Table 2. Participants that started the DCP (*n* = 91), defined as performing at least one sensor-guided workout, performed an average of 36 in-app workouts, or 3.0 workouts per week from week 1 to week 12. Users that completed the outcome questionnaires at week 12 (n = 69) performed 45 sensor-guided workouts (3.8 workouts per week), compared to the 3 times per week recommended in the DCP. Average weekly engagement, defined as any progress towards the weekly goals, was 75% for those that started the program, and 90% for those that completed the program. Participants that completed the week 12 follow-up read 9.2 education articles, completed 1.7 cognitive behavioral therapy sessions, and posted on the feed 6.3 times. Participant engagement levels met or exceed all goals set by the program.

**Table 2:** Engagement indicators for each of the aspects of the DCP. “Starters” indicates participants performed at least one sensor-guided workout. “Finishers” indicates participants that completed the outcomes questionnaires at 12-week follow-up. SD: standard deviation

	All starters (*n* = 91)	Finishers (*n* = 69)
Number of workouts, mean (SD)	35.7 (28.9)	44.8 (26.7)
Users engaging with the program per week, % (*n*)	75%	90%
Users active with sensor-guided exercise in weeks 1–4, %	90%	99%
Users active with sensor-guided exercise in weeks 5–8, %	77%	94%
Users active with sensor-guided exercise in weeks 9–12, %	68%	87%
Offline activities logged in hours, mean (SD)	12.1 (12.5)	15.3 (12.5)
Education articles read, mean (SD)	7.4 (4.4)	9.2 (3.3)
Cognitive Behavioral Therapy session completed, mean (SD)	1.4 (1.2)	1.7 (1.1)
Team posts and comments, mean (SD)	4.9 (4.7)	6.3 (4.6)

### Primary and secondary outcomes

**Fig. 2:**
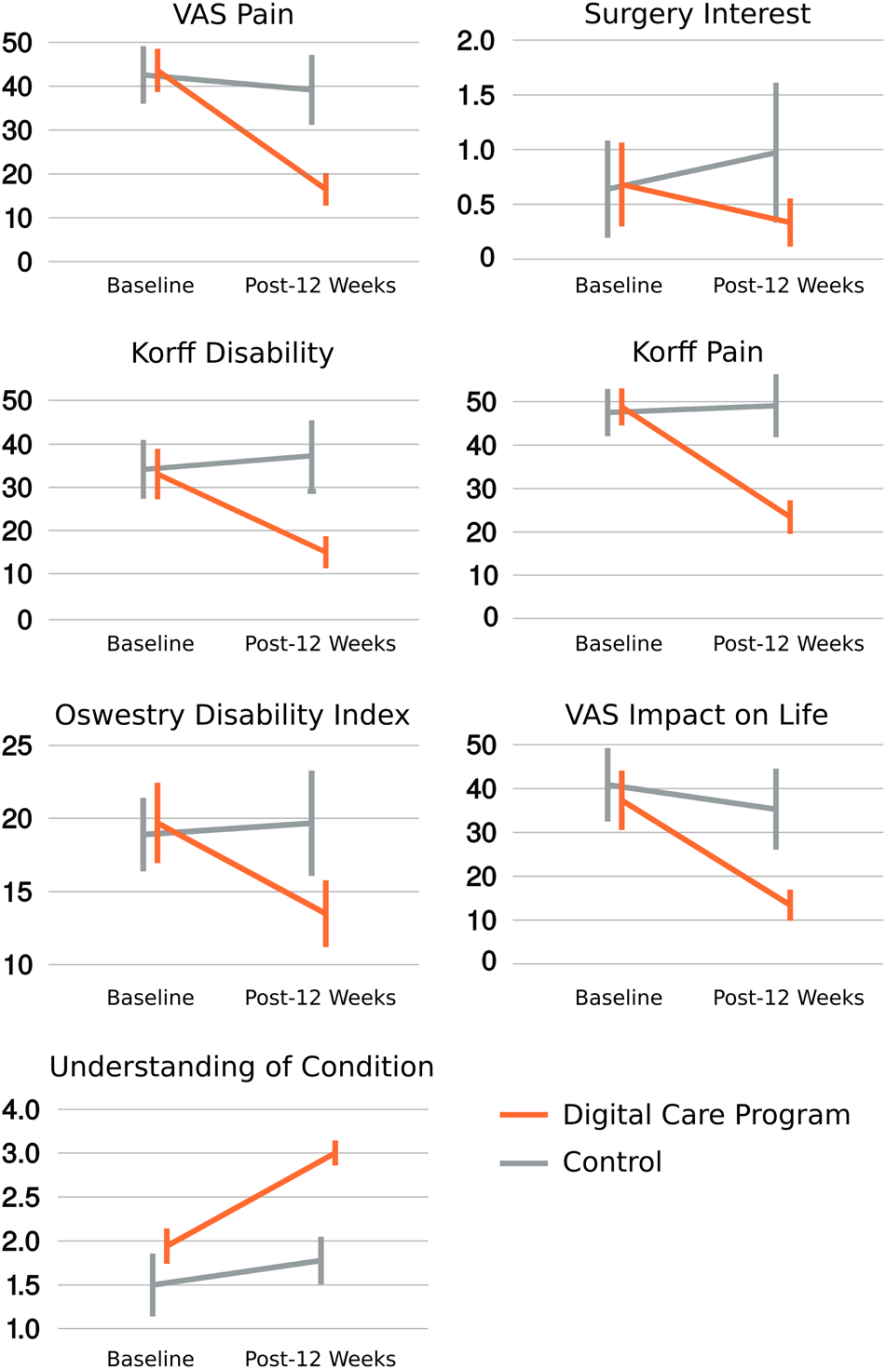
All per protocol primary and secondary outcomes visualized. Korff, Oswestry disability index, and visual analog scale (VAS) outcomes are on scales from 0 to 100; surgery interest is on a scale from 0 to 10; and understanding of condition is on a scale from 0 to 4. Error bars represent 95% confidence intervals

The intention-to-treat results in Table 3 and Fig. 2 show participants on the DCP experienced statistically significant greater improvements at week 12 on all primary and secondary outcomes compared to the control group. The conservative intention-to-treat analysis - in which every randomized participant is included irrespective of completion - shows the DCP’s causal effect on participants’ wellbeing as measured in LBP (primary outcome: Korff Pain; secondary outcome: VAS pain), disability (primary outcomes: Korff Disability, Oswestry Disability Index; secondary outcome: VAS Impact on Daily Life), as well as secondary outcomes of understanding of LBP and reduction in back surgery interest.

**Table 3:** Primary and secondary outcomes. Results are listed for both the intention-to-treat group, which includes subjects who did not start or complete the 12-week program (ITT), as well as per protocol results for subjects that completed 12-week program (PP)

		DCP at baseline, mean (SD)	DCP at outcome, mean (SD)	Control at baseline, mean (SD)	Control at outcome, mean (SD)	Group difference, mean (95% CI)	Group difference, *p*-value
*Primary outcomes*							
Korff pain	ITT	51.1 (17.8)	33.8 (21.6)	51.4 (17.4)	50.5 (21.4)	−16.4 [−22, −10.9]	<0.001
	PP	48.8 (17.8)	23.4 (16.1)	47.5 (16.1)	49.1 (21.4)	−26.9 [−33.8, −20]	<0.001
Korff disability	ITT	34.3 (23.1)	21.5 (19.6)	40.3 (24)	40.5 (25.7)	−13 [−19.3, −6.7]	<0.001
	PP	33.1 (24.3)	15 (15.5)	34.2 (20.2)	37.3 (24.3)	−21.3 [−30.8, −11.7]	<0.001
ODI	ITT	21.7 (12.1)	17.6 (12)	21 (9.66)	21.1 (11.2)	−4.1 [-6.5, −1.8]	<0.001
	PP	19.7 (11.4)	13.5 (9.46)	18.9 (7.4)	19.7 (10.6)	−6.9 [−10.5, −3.3]	<0.001
*Secondary outcomes*							
VAS Pain score	ITT	46.3 (20.9)	25.8 (21.4)	45.4 (20.8)	40.8 (23.2)	−16 [−22.5, −9.4]	<0.001
	PP	43.6 (20.5)	16.5 (15.5)	42.6 (19.4)	39.2 (23.6)	−23.7 [−31.9, −15.5]	<0.001
VAS impact on daily life score	ITT	38.6 (26.6)	21.1 (20.7)	43.9 (25.2)	38.2 (26.1)	−11.8 [−19.3, −4.3]	0.002
	PP	37.3 (28.2)	13.4 (14.8)	40.9 (24.7)	35.3 (27.3)	−18.3 [−29, −7.7]	0.001
Surgery interest	ITT	0.894 (1.71)	0.619 (1.35)	1.39 (2.55)	1.53 (2.67)	−0.4 [−0.7, −0.1]	0.01
	PP	0.681 (1.59)	0.333 (0.918)	0.639 (1.31)	0.972 (1.89)	−0.7 [−1.2, −0.2]	0.006
Understanding of condition and treatment options (0-4)	ITT	1.81 (0.95)	2.47 (1.07)	1.77 (1.03)	1.94 (0.871)	0.5 [0.2, 0.7]	0.0005
	PP	1.94 (0.838)	3 (0.594)	1.5 (1.06)	1.78 (0.797)	0.8 [0.4, 1.2]	0.0001

All *p*-values are from two-sided statistical tests

*SD* standard deviation, *ODI* Oswestry Disability Index, *VAS* visual analogue scale

The intention-to-treat analysis in Table 3 shows the *average* benefit of the program on all those that were randomized, irrespective of whether they withdrew before even starting or finishing the DCP. As such, the intention-to-treat analysis underestimates the benefit of the program for those that complete the program. The per protocol analysis in Table 3 demonstrates that participants who completed the DCP experienced greater benefits across all outcomes compared to those that completed the control arm. For example, VAS pain dropped 62%, from 43.6 to 16.5 on a scale from 0 to 100, in those that completed the DCP, compared to an 8% reduction in the control group.

Finally, we also examined what proportion of per protocol participants reached a minimally important change (MIC) in their ODI and VAS pain scores. The MIC is 10 points for ODI, 15 points for VAS, or 30% of baseline.^23^ Table 4 shows that participants in the treatment group are significantly more likely to achieve MIC compared to the control group for both ODI and VAS, irrespective of how the MIC is defined precisely. The only exception is the ODI MIC of 10 points, which was achieved by 28% of treatment and 11% of control participants (*p* = 0.09). This can be attributed to the low number of participants in the control group, whereas a test of proportions requires greater sample size than the regression models used in Table 3. Overall, 81% of treatment participants achieved MIC on VAS either as expressed by absolute or percentage improvement, compared to 31% in the control group (p < 0.001). Similarly, 58% of the participants receiving the digital care program reached either MIC for ODI compared to 25% in the control group (*p* = 0.003).

**Table 4:** Proportion of participants reaching a minimal important change (MIC) in the per protocol group. The MIC are taken from Ostelo et al. (2008) and represent a change in the VAS or ODI score, respectively, that is considered meaningful to the participant. It is defined as either a 15/10 absolute point change for VAS/ODI, or as a 30% reduction from the baseline score. We also show how many participants reached either of the MIC definitions (last two rows). The *p*-values show the outcome of a two-sided test of proportions between treatment and control, revealing a larger proportion of participants in treatment achieved MIC than in control

MIC achieved for outcome	Treatment	Control	*p*-value for test of proportions
VAS, 15-point reduction	48/69 (70%)	8/36 (22%)	<0.001
ODI, 10-point reduction	19/69 (28%)	4/36 (11%)	0.09
VAS, 30% reduction	56/69 (81%)	10/36 (28%)	<0.001
ODI, 30% reduction	38/69 (55%)	9/36 (25%)	0.006
VAS, absolute OR percentage reduction	56/69 (81%)	11/36 (31%)	<0.001
ODI, absolute OR percentage reduction	40/69 (58%)	9/36 (25%)	0.003

## Discussion

Results from this RCT assessing the efficacy of a 12-week DCP for LBP found subject-reported pain and disability significantly improved compared to a control group undergoing treatment-as-usual. Results also demonstrated a reduced interest in pursuing LBP surgery following the DCP, which is likely attributable to the reduced reported pain and disability. Improved outcomes were observed in the context of a comprehensive approach involving conservative therapies for LBP as well as strong engagement of participants. The positive results from this work support the potential for using a DCP as a treatment for the large number of individuals with LBP that medical experts recommend receive non-invasive therapies before drugs or surgery.

Analysis of subject-reported pain and disability demonstrated significant improvement in all related outcomes for DCP group compared to control group. When considering participants who completed the study per protocol, the improvement in Korff pain, VAS pain, and its impact on daily life were 52%, 62%, and 64% for the DCP treatment group compared to 3%, 3 and 9% for the control group. Similarly, the per protocol improvement in Korff disability and Oswestry Disability Index were 55 and 31% for the DCP treatment group compared to 9 and 4% for the control group. Oswestry Disability Index is a widely used metric of LBP disability. Despite the study’s baseline Oswestry Disability Index scores being low, we observed improvements that fell within the range of Minimally Important Change.^23^ Based on other clinical studies, the DCP could have a greater impact on subjects with more severe and longer-lasting LBP.^9,24–26^

Participants’ understanding of condition and treatment options demonstrated a 55% improvement for the DCP compared to 19% for control group in a per protocol analysis. Critically, the DCP treatment group showed a 52% decrease in average interest in surgery while the control group showed a 53% increase in average interest in surgery. Although baseline values for surgical interest were low for our study population, we anticipate—and have confirmed this in an unpublished follow-up study—that surgical interest would be similarly reduced for a sample with more severe LBP symptoms and chronicity. Confirming long-term reductions in surgery utilization following the DCP will be important, as LBP presents a large economic burden throughout the world. Compared to non-invasive treatment options, the costs associated with spinal surgery are significantly greater and have been shown to deliver no better outcomes.^27,28^ The positive impact from the DCP on LBP demonstrates potential for both direct and indirect cost savings by avoiding surgery and regaining function in daily life. Beyond potentially avoiding invasive surgical interventions for LBP, the effectiveness of a DCP may mitigate the rising use of harmful opioids for coping with LBP. The assumed impact of the DCP on reducing missed work days and reliance on opioids as a treatment for LBP were not included in the present study and will be assessed in future studies using clinical populations with a higher prevalence of these characteristics.

The success of any non-invasive therapy to impact clinical outcomes requires patients to actually use and engage with the treatment. Typical clinically administered non-invasive care approaches have shown promising but inconsistent results on LBP patient outcomes.^9^ Interestingly, results from the few prior RCTs on digital programs for self-management for LBP are mostly inconclusive regarding effectiveness.^14^ Beyond the variability in the types of intervention and outcomes assessed among prior studies, another source of inconsistent and unconvincing results may be due to inadequate patient adherence to the conservative therapies.

A strength of using a digital program for disease management is the potential to enhance patient involvement in their recovery process and outcomes. A recent study of a mobile app delivering multidisciplinary treatment for pain related to LBP found the app had a positive effect on VAS pain, but compared to our study, they reported both a lower improvement in VAS pain and lower percentage of subject participation over the 4- to 12-week treatment period.^29^ Although some percentage of subject dropout is expected, at the 12-week point Huber et al. retained only 17% of participants compared to our DCP retaining 76% of participants that started the program. We attribute our higher retainment of participants to elements of the DCP aimed at enhancing user engagement, including health coaching, peer group interaction, weekly checklists, and points goals, each of which could be adjusted to the individual needs of the user Beyond the benefits of enhanced participation with the program, patient engagement is critical for the success of these digital applications for self-managing LBP because it aids the development of healthy habits and routines that successfully manage LBP. Studies show that greater self-management competency^30^ and greater adherence to rehabilitation exercise programs^31^ are associated with a stronger sense of internal control during a patient’s musculoskeletal care process.

The DCP evaluated here is built to maximize patient engagement by providing unhindered access to tailored content and real-time feedback through sensors and coaching. Participants were able to complete the entire program through a tablet app at home or anywhere else, whether or not they had wireless internet available. The weekly checklist of actions could be completed at any time of day, in whichever order was most convenient. A dedicated health coach provided unlimited behavioral coaching via telephone, email, in-app message, and text message to provide support or encouragement during periods of decreased engagement, symptom flares, general questions or technical issues. In contrast, in traditional LBP care participants have to spend significant time traveling to their care provider at specific times in the week; they have to manage multiple providers for different services (e.g. physical therapy exercises, cognitive behavioral therapy); the care provider is not usually on standby for questions or issues that come up during the day; and critically, the care provider has no way of monitoring daily patient engagement and wellbeing.

Participants engaged well with the program. Though we observed drop-off after randomization (19%) for participants who filled out the online screener but did not continue to the DCP, 76% of participants that started the DCP completed their assessment after 12 weeks and in an average week of the DCP, 90% of them engaged with the program. Though no conclusive evidence is reported here, we attribute this strong engagement to a personalized experience for participants. They could complete the tasks set out at their convenience, with weekly checklists and points goals, as well as a professional health coach and peer group to keep participants accountable. Novel content was introduced throughout the 12 weeks, with users unlocking new education, and new sensor-guided exercises. We recommend others developing DCPs to consider the user’s desire for diverse novel content and convenience. There is an opportunity to continue to further personalize the participant DCP experience by leveraging artificial intelligence to optimize recommendations. Furthermore, a DCP has the potential to scale delivery of evidence-based recommended care to the ever-growing worldwide number of LBP patients.

When interpreting the results of this study, its strengths and limitations should be considered. Strengths of this study include the randomized controlled study design, and that the study was designed, conducted, and analyzed according to a pre-specified protocol. Also, the digital format of the program provides flexibility and convenience for users, supporting adherence to the program. One possible limitation includes that the treatment in this RCT was non-blinded and while this is common within the field, it prevents us from knowing whether the effect of the trial may be in part due to the participant’s expectation that their symptoms would diminish as they were assigned to the treatment. A second concern is due the remote nature of the program, participants were not assessed by a clinician, their medical records were not evaluated, and generally there was little coordination with the conventional healthcare system. However, LBP is commonly diagnosed through self-report as done in this study. We also used standard questionnaires to screen for any complaints that may indicate specific conditions (red flags) and referred those to healthcare professionals. This DCP was designed so participants could independently seek traditional care if desired. Future studies will investigate the effect of the DCP on a clinical cohort and clinical status of participants will be followed.

Another concern is that participants in the control group would exaggerate their week-12 symptoms in an attempt to gain preferential access in the next round of the program. Though this is a possibility, we hoped to deter participants from overstating their symptoms by guaranteeing participation in the following round for those initially placed in the control group. Additionally, if overstating symptoms was widespread, we would have expected scores across the board to substantially increase at week 12, however the per protocol scores either decreased or increased by a few points at most. Finally, while this study found significant improvements in primary and secondary outcomes associated with the DCP, a last limitation is that our trial did not investigate outcomes beyond the first 12 weeks. Next steps include studies on outcomes from multi-year follow-up.

In conclusion, this RCT shows that care provided using a DCP substantially reduces pain, disability, and surgery interest in those living with LBP. In the per protocol DCP treatment segment, we found strong patient engagement. Care from the DCP was achieved through a program that was delivered remotely, using technology that has the potential to scale evidence-based conservative care to an ever-growing worldwide number of LBP patients.

## Methods

### Study design

This study was a two-armed, randomized, controlled, unblinded trial of participants with chronic non-specific LBP. Employees and their dependents at participating employers, across 12 locations in the US, were invited to complete an online application. Employees were highly diverse, and included both office and service based roles such as data analysts, drivers, catering staff, and outdoor instructors. Participants were recruited through emails, direct mail, and posters between January and March, 2017. The trial was approved by the Western Institutional Review Board and we have complied with all ethical regulations. Participants provided informed consent and completed the intervention at home. The trial was preregistered at International Standard Randomized Controlled Trial Number (ISRCTN) 42338218. We followed CONSORT guidelines for reporting this trial.

### Study population

We assessed the eligibility of all applicants that completed the baseline questionnaire for LBP through a web-based questionnaire. Participants provided informed consent as part of this questionnaire by ticking a checkbox after reading the digital information sheet. The inclusion criteria were: (1) age over 18 years, (2) non-specific LBP for at least 6 weeks in the past 12 months, (3) participating in the collaborating employers’ health plans, and (4) provision of informed consent. The exclusion criteria were (1) surgery on the back less than 3 months ago, (2) injury to the back less than 3 months ago, (3) did not indicate ‘lower back’ when asked about pain location. As there were a limited number of places available on the program, eligible applicants were prioritized for enrollment, with those exhibiting greater pain, disability, and surgery intent prioritized over those showing less. Applicants not selected for the study were placed on a waitlist for future deployments at the same site outside of the scope of the trial. Participants were not paid for their time, other than an incentive offered to complete the outcome questionnaire for those participants that did not complete it within 4 days of first invitation. No harm was observed or reported in either arm of the experiment.

### Randomization

Applicants were randomized into the trial twice weekly during the signup period by randomizing batches of participants into treatment and control using a 60:40 treatment-to-control ratio (*n* = 128) or using an 80:20 ratio (*n* = 49). The 80:20 ratio was used for a restrictive period of time due to administrative error. The effective allocation ratio was therefore 64:36 treatment-to-control. When a batch of applicants was randomized, an algorithm with random seed shuffled the batch and selected the first 60% to enter the treatment, and the remaining 40% to enter control. The person reviewing the applicants had no way of knowing whether any given applicant would enter treatment or control (concealed allocation). After randomization, participants in the treatment group received an email inviting them to complete their profile and received the kit to participate in the DCP, whereas those in the control group received an email with three education articles to help them care for their back. Due to the nature of the study, neither the study staff nor the participants were blinded to group allocation.

### Study intervention

The treatment group received a 12-week DCP for LBP developed by physical therapists, medical doctors, engineers, and subject-matter experts at a digital health company. Participants received a tablet computer with the DCP app installed, and two bluetooth wearable motion-sensors with straps to be placed along the lower back and torso during the in-app exercise therapy. Participants were assigned a personal coach that provided unlimited support and accountability throughout the program and were placed in a team to provide peer support through a discussion feed within the app. All app participation was completed remotely, at times and places chosen by the participant. Each week, participants in the DCP were instructed to complete 3 sessions of sensor-guided physical exercise, read 1 to 2 education articles, log their symptoms at least twice, perform cognitive behavioral therapy on a subset of weeks, and track a recommended 3 aerobic activities per week. Each participant also maintained access to treatment as usual.

The control group received three digital education articles from the DCP. These articles discussed the importance of self-care, how to deal with setbacks in LBP, and how to manage communication and relationships when living with chronic LBP. The control group maintained access to treatment-as-usual and were informed that they would be reconsidered for the program when enrollment reopened after the 12-week study.

The 12-week program received extensive testing over a 2-year period prior to starting the trial. All participants received the same version of the program, and there were no major app updates during the course of the trial.

### Study outcomes

#### Primary outcomes

Participants completed the Modified Von Korff (MvK) scales^32^ at screening and at week 11 (control group), or screening, week 4, week 8, and week 11 (treatment group). The MvK yields a pain and a disability metric, both from 0 (minimum) to 100 (maximum). The third primary outcome was the Oswestry Disability Index^33^ (ODI) which falls between 0 (no disability) to 100 (complete disability). The ODI was collected at baseline and week 11 for both treatment and control groups. To conclude a positive effect of treatment we required a significant effect on all three primary outcomes, though we note this was not specified in the preregistration.

#### Secondary outcomes

First, a visual analogue scale (VAS) for the question “Over the past 24 h, how bad was your back pain?” from 0 (none) to 100 (worst imaginable). Second, a VAS for the question “Over the past 24 h, how much has back pain interfered with your daily activities?” from 0 (none) to 100 (worst imaginable). Thirdly, we assessed surgery intent using the question “On a scale of 0 to 10 how interested are you in back surgery?” with labels “not at all” at 0, and “definitely going to get surgery” at 10. Lastly, we asked “Thinking about your symptoms, how well do you feel you understand your condition and your treatment options?” with answers “Not at all”, “Slightly”, “Moderately”, “Very well”, “Completely”, coded from 0 to 4. All data were assessed at baseline and at the end of the 12-week DCP in both the treatment and control groups. Additionally, those in the treatment group were asked to complete these questions at various points during the DCP: the VAS twice each week, and the questions related to surgery and understanding of their condition at week 6.

#### Sample size

We assessed the required sample size to detect a difference in change of 10 points on the 100-point MvK pain scale, with a standard deviation of 20 based on past experience with the questionnaire. The number of participants needed in each group to detect a 10-point difference given a Type I error rate of 0.05 and power of 0.8 was calculated. Given our unequal allocation ratio, we would need at least 79 in the treatment group and 53 in the control group for a total of at least 132 participants in the trial. We opted for an unequal allocation ratio to ensure we would be able to enter a certain minimum number of people into the treatment arm, a criterion mandated by the commercial nature of the deployments.

#### Statistical analyses

Our primary analysis was conducted using an intent-to-treat approach. This analysis included all participants that were randomized, including those in the treatment group that never started the DCP, as well as those in control that were enrolled in the DCP by accident. We describe baseline characteristics for the treatment and control groups based on the screening questionnaire. We also describe metrics of engagement (not a registered outcome) with the DCP for two groups of participants: those in the treatment group that performed at least one session of exercise therapy, and those that completed the week 12 outcome questionnaire. The analysis of preregistered primary and secondary outcomes was performed using a linear mixed model using the “lme4” package^34^ in R with factors “time point” (baseline or outcome) and “group” (treatment or control) and their interaction. We modeled a separate baseline for each participant, effectively examining the change scores only (in lme4 this was performed as “score~timepoint*group + (1|participant)”, where (1|participant) models an intercept for each participant separately). We assessed normality of the residuals based on quantile-quantile (QQ)-plots. If we did not have outcome data for a participant, we used last observation carried forward (LOCF). We also analyzed all primary and secondary outcomes with baseline carried forward (BOCF) also for the treatment group (rather than LOCF). We also omitted LOCF and instead allowed the mixed-effects model to account for the missing data, which yielded an identical pattern of results as using LOCF and BOCF. We also report results for a per protocol analysis to assess the effect of the program on those that completed it. All *p*-values are from two-sided tests.

### Code availability

Analysis code is available on request due to privacy or other restrictions. All code was written in R version 3.x.

## Data Availability

Data are available on request due to privacy or other restrictions.
